# Ionic liquid-assisted synthesis of chitin–ethylene glycol hydrogels as electrolyte membranes for sustainable electrochemical capacitors

**DOI:** 10.1038/s41598-022-12931-w

**Published:** 2022-05-25

**Authors:** Marcin Wysokowski, Krzysztof Nowacki, Filip Jaworski, Michał Niemczak, Przemysław Bartczak, Mariusz Sandomierski, Adam Piasecki, Maciej Galiński, Teofil Jesionowski

**Affiliations:** 1 Institute of Chemical Technology and Engineering, Faculty of Chemical Technology, Poznan University of Technology, Berdychowo 4, 60965 Poznan, Poland; 2 Institute of Chemistry and Applied Electrochemistry, Faculty of Chemical Technology, Poznan University of Technology, Berdychowo 4, 60965 Poznan, Poland; 3 Institute of Materials Engineering, Poznan University of Technology, Piotrowo 3, 61138 Poznan, Poland

**Keywords:** Sustainability, Electrochemistry, Materials chemistry, Gels and hydrogels

## Abstract

A novel chitin–ethylene glycol hybrid gel was prepared as a hydrogel electrolyte for electrical double-layer capacitors (EDLCs) using 1-butyl-3-methylimidazolium acetate [Bmim][Ac] as a chitin solvent. Examination of the morphology and topography of the chitin–EG membrane showed a homogeneous and smooth surface, while the thickness of the membrane obtained was 27 µm. The electrochemical performance of the chitin–EG hydrogel electrolyte was investigated by cyclic voltammetry and galvanostatic charge/discharge measurements. The specific capacitance value of the EDLC with chitin–EG hydrogel electrolyte was found to be 109 F g^−1^ in a potential range from 0 to 0.8 V. The tested hydrogel material was electrochemically stable and did not decompose even after 10,000 GCD cycles. Additionally, the EDLC test cell with chitin–EG hydrogel as electrolyte exhibited superior capacitance retention after 10,000 charge/discharge cycles compared with a commercial glass fiber membrane.

## Introduction

Chitin is a linear polymer, the second most abundant polysaccharide (after cellulose) on Earth. Nature generates approximately 100 billion tonnes of chitin annually, and the substance can be isolated from a wide variety of sources^1–3^. These include fungi, plankton, sponges, and the exoskeletons of insects and crustaceans^4^. Every year, around 6–8 million tonnes of chitin-rich seafood waste (in the form of crab, shrimp, and lobster shells) are produced worldwide^1^. The current system of seafood processing leads to the accumulation of many waste products and has become very problematic, especially in some developing countries. Therefore, increased attention should be paid by governments, industry, and academia to the development of new sustainable pathways to refine crustacean shells as an abundant and cheap renewable resource^1,5,6^. Logically, in response to these needs, scientific work should focus on developing new chitin-based materials or finding novel applications for this biopolymer^7^.

To meet the recent demands of sustainable chemistry and strict environmental regulations, supercapacitor technology is becoming focused on the potential use of renewable materials in the design of energy storage devices^8–11^. Recently, polysaccharides have become attractive options for the fabrication of solid-state or hydrogel electrolytes in electrochemical double-layer capacitors (EDLCs). The electrolyte, as one of the most important components of an EDLC, is subject to continuous optimization aimed at improving the electrochemical characteristics of the device. The main innovations developed to increase the speed of the charging/discharging process and to reduce the internal resistance in the capacitor (elimination of the passage of electrolyte ions through the separator) are solid or quasi-solid-state (gel and hydrogel) polymer electrolytes^12^. Quasi-solid-state polymer electrolytes (GPEs) are described as solid polymer matrices with a liquid electrolyte trapped inside the polymer chain interspaces. When the liquid electrolyte within the polymer matrix is based on water, a GPE can be called a hydrogel electrolyte^13^. Among all quasi- and solid-state electrolytes used in EDLCs, hydrogels have been investigated relatively poorly, despite their unique characteristics that combine the advantages of solid-state and liquid electrolytes. The synergistic effect within these materials combines the benefits of a solid-state electrically inert, porous, and ion-permeable separator with the high ionic conductivity characteristic of liquid electrolytes based on water^14^. To form the hydrogel electrolyte, the polymer matrix has to be electrically and chemically inert, flexible, and durable. However, above all, it must be a material with a complex 3D structure of polymer chains and hydrophilic properties, accompanied by the ability to swell^15^. These conditions are fulfilled by biobased polysaccharides, which also offer three essential advantages over synthetic polymers. Polysaccharides, such as cellulose, chitin, chitosan, and alginates, are promising hydrogel materials for electrochemical applications not only because of their unique 3D polymeric structure, but most of all because of their biodegradability, nontoxicity, and modification potential^15–18^. This explains why polysaccharides are coming to be seen as attractive for the fabrication of hydrogel electrolytes, and how their application in electrochemical energy storage devices (such as EDLCs) can reduce the release of hazardous substances into the environment and enable the production of more flexible and thinner devices^13,19^. Previously, chitosan-based membranes were reported as hydrogel electrolytes for EDLCs^20^. However, the mechanical properties of the material were not satisfactory, and reinforcement was required. Reinforcement with a naturally prefabricated scaffold isolated from marine sponges resulted in better mechanical resistance and improved specific capacitances. The full potential of chitin in the development of sustainable EDLCs is still poorly explored. This is associated with chitin’s exceptional chemical stability, due to the formation of a network of strong inter- and intra-fibrillar hydrogen bonds that make the processing of chitin difficult^11,21,22^. Chitin is isolated in the form of flakes or powder, and due to its chemical resistance, it requires various treatments to process it into a form tailored to a specific application. On the other hand, the high chemical stability of chitin is the main advantage of the use of this biopolymer as a polymer matrix for the hydrogel electrolyte in electrochemical capacitors, ensuring proper resistance in various liquid electrolytes^23,24^. Technological development has equipped scientists with new tools and methods for processing this polymer into desired forms^25–31^. For instance, acidic hydrolysis^32^, TEMPO-mediated^33^ and peroxide-mediated oxidation methods^34^ or a freeze-thawing process^35^ are often applied for the preparation of chitin-based membranes. Nevertheless, these methods require the use of harsh chemicals, and hydrolyzed chitin nanocrystals or nanowhiskers often have an unfavourably low length/diameter ratio. Therefore, the utilization of ionic liquids and deep eutectic solvents seems to be an important milestone that opens new perspectives for chitin processing and the development of a new generation of chitin-based materials enabling this biopolymer to fulfil its promise. Both types of solvents are classified as forms of green biomass processing and comply with the principles of sustainable chemistry and engineering. The application of ionic liquids allows the fully controlled fabrication of chitinous membranes, films, nanofibrils or even 3D printed scaffolds with unique physicochemical properties attractive for various sophisticated applications^36–38^. Moreover, ionic liquids can be easily recovered and reused for chitin processing^37^. An important benefit of ILs is that true dissolution of chitin instead of nanofibrilation can generate a significant number of task-specific derivatives with tailored functionalities^39,40^. ILs also play an important role in the design of novel energy storage devices—for review please see^41^ .

We hypothesize here that α-chitin blended with ethylene glycol could form a membrane with mechanical and electrochemical properties attractive for green and sustainable EDLCs. Thus, the study’s main goal was to determine, for the first time, the potential utility of a chitin-based film, prepared from IL solutions and plasticized with ethylene glycol, as a polymer matrix for the hydrogel electrolyte in an EDLC. The morphological and electrochemical properties of the obtained materials were determined with modern analytical tools, and their electrochemical performance was compared with that of a commercial synthetic separator.

## Methods

All experiments described here used α-chitin isolated from snow crabs, in the form of fine powder with particle size < 200 µm, molecular weight > 2000 kDa and acetylation degree > 80% (Heppe Medical Chitosan GmbH, Germany). 1-Methylimidazole (purity 99%), 1-chlorobutane (purity 99%), acetic acid (purity 99%) and Dowex Monosphere resin 550A (OH) were obtained from Sigma-Aldrich (St. Louis, MO, USA). All solvents (methanol of purity 99.5%, acetonitrile of purity 99.5%, ethyl acetate of purity 99%) were purchased from Avantor (Gliwice, Poland) and used without further purification.

### Synthesis of 1-butyl-3-methylimidazolium acetate [Bmim][Ac]

The first step involved the synthesis of 1-butyl-3-methylimidazolium chloride. In a 250 ml round-bottomed flask equipped with a Teflon-coated magnetic stirring bar, 1-methylimidazole (0.10 mol) was reacted with 1-chlorobutane (0.11 mol) added at once to 100 ml of acetonitrile, which was utilized as a reaction medium. The reactants were stirred for 48 h at 60 °C, and the solvent was then evaporated using a vacuum evaporator. The product was washed twice with 100 ml of ethyl acetate to remove excess of quaternizing agent. The remaining ethyl acetate was removed by drying the content of the flask under reduced pressure at 50 °C for 24 h. Subsequently, 0.10 mol of 1-butyl-3-methylimidazolium chloride was dissolved with 200 ml of methanol in a 500 ml reaction glass equipped with a mechanical stirrer. Then, 100 ml of the anionic resin Dowex Monosphere 550A (OH) was added, and the obtained mixture was stirred for 1 h at 25 °C. The resin was then filtered off and rinsed three times with small amounts of methanol. The solution of the semi-product 1-butyl-3-methylimidazolium hydroxide was slowly neutralized using stoichiometric amounts of acetic acid at 25 °C in an EasyMax 102 (Mettler Toledo, Switzerland) semi-automated reactor system equipped with a glass electrode. The solvent was evaporated under vacuum in a rotary evaporator, and the product was dried under reduced pressure (5 mbar) at 50 °C for 24 h. The structure of the obtained compound was confirmed by analysis of the collected FT-IR, ^1^H, and ^13^C NMR spectra (Figures S1–S3, ESI). The water content in the ionic liquid, assessed with the use of an SI Analytics Titroline 7500 KF Trace coulometer, was determined to be 6015 ppm.

^1^H NMR spectra were recorded on a Varian VNMR-S 400 MHz spectrometer with TMS as internal standard. ^13^C NMR spectra were obtained with the same instrument at 100 MHz. The IR spectra were collected using the EasyMax 102 semi-automated system (Mettler Toledo, Switzerland) connected to a ReactIR iC15 probe (Mettler Toledo) equipped with an MCT detector and a 9.5 mm AgX probe with a diamond tip. Data were sampled from 3000 to 650 cm^−1^ with 8 cm^−1^ resolution and processed with the use of iCIR 4.3 software.

### Fabrication of chitin–ethylene glycol thin films

Chitin dissolution was carried out using the EasyMax 102 system (Mettler Toledo, Switzerland), which allows the high-precision control of a broad range of reaction parameters such as temperature, pH, stirring speed, and reagent addition, according to a previously reported protocol^42^. Briefly, 0.1 g of α-chitin and 10 ml of 1-butyl-3-methylimidazolium acetate [Bmim][Ac] along with a magnetic stir bar were placed in a 20 ml vial, heated to 95 °C and stirred at 1000 rpm for 24 h until the chitin was fully dissolved and the solution became homogeneous and turned an amber-like color. The solution was additionally centrifuged to remove any undissolved residuals. To prepare the thin film, 2 ml of hot solution was poured over a Teflon-lined Petri dish. Once the solution had settled and formed a uniform layer (30 min), the Petri dish was placed in an ethylene glycol (EG) bath for 48 h to allow film coagulation. During this time, the ethylene glycol was changed four times, to ensure the complete removal of the ionic liquid. Subsequently, the gel formed was immersed in a distilled water bath for 24 h to remove excess ethylene glycol, and the distilled water was changed four times. The resulting chitin–EG films were then air-dried for 72 h, and a detailed analysis was made of their physicochemical and electrochemical parameters.

### Scanning electron microscopy

A MIRA3 scanning electron microscope (SEM) (Tescan, Czech Republic) was used to assess the surface. This was done using an acceleration voltage of 2 kV and a working distance of 2 mm. A thin Au–Pd layer with a thickness of approximately 10 nm was deposited on the sample using a Quorum vacuum evaporator.

### Atomic force microscopy

The nanomechanical properties of the chitin–EG membrane were determined with the use of atomic force microscopy (AFM). This technique enables the creation of three-dimensional images of the surface of a sample. An NX10 microscope (Park Systems) was used in the experiment.

To find a suitable point on the surface, initial images were made in non-contact mode (NCM) with a 30 × 30 μm scan size. Later, the scan size was narrowed to 10 × 10 μm. Mechanical properties of the surface were examined in PINPOINT™ mode, which additionally allows the simultaneous collection of data on six mechanical properties: adhesion energy, adhesion force, deformation, energy dissipation, Young’s modulus, and stiffness.

Samples were made by attaching the chitin–EG membrane to a steel puck with the use of double-sided adhesive tape. The cantilever used in the measurements was an ALL-IN-ONE D (BudgetSensors), which has a nominal force constant of 40 N/m. The surface area examined was 10 × 10 μm, scanned with 512 lines per image and with the NCM mode scan speed set at approximately 0.3–0.4 Hz. All measurements were performed at room temperature (approximately 22 °C). The resulting data were investigated using the open-source Gwyddion software and XEI (Park Systems) AFM analysis programs.

### Contact angle measurements

The wettability and surface free energy of the prepared chitin–EG thin films were determined by static contact angle measurements, with distilled water and diiodomethane as the polar and dispersion components, respectively. Measurements were made using a sessile drop technique at 25 °C with a DSA100 goniometer (KRÜSS, Germany). The surface free energy was calculated using the Owens, Wendt, Rabel & Kaelble (OWRK) model.

### Swelling factor

The swelling factor of the chitin–EG membranes was evaluated by determining their sorption of 2 M aqueous solution of lithium acetate. The dry chitin–EG was cut into 1 cm × 1 cm fragments, which were weighed to determine their dry weight. These samples were placed in 50 ml beakers containing 40 ml of 2 M lithium acetate solution, and stored at 25 °C. After 24 h the samples were taken out and weighed after carefully removing their surface water with a filter paper. The swelling was calculated using Eq. (1)^43^:

1 $$SF=\frac{{m}_{w}-{m}_{d}}{{m}_{d}}*100\mathrm{\%}$$

where *m*_*w*_ is the mass of the wet sample and *m*_*d*_ is the mass of the dry sample.

### Ionic conductivity measurements 

**Figure 1:**
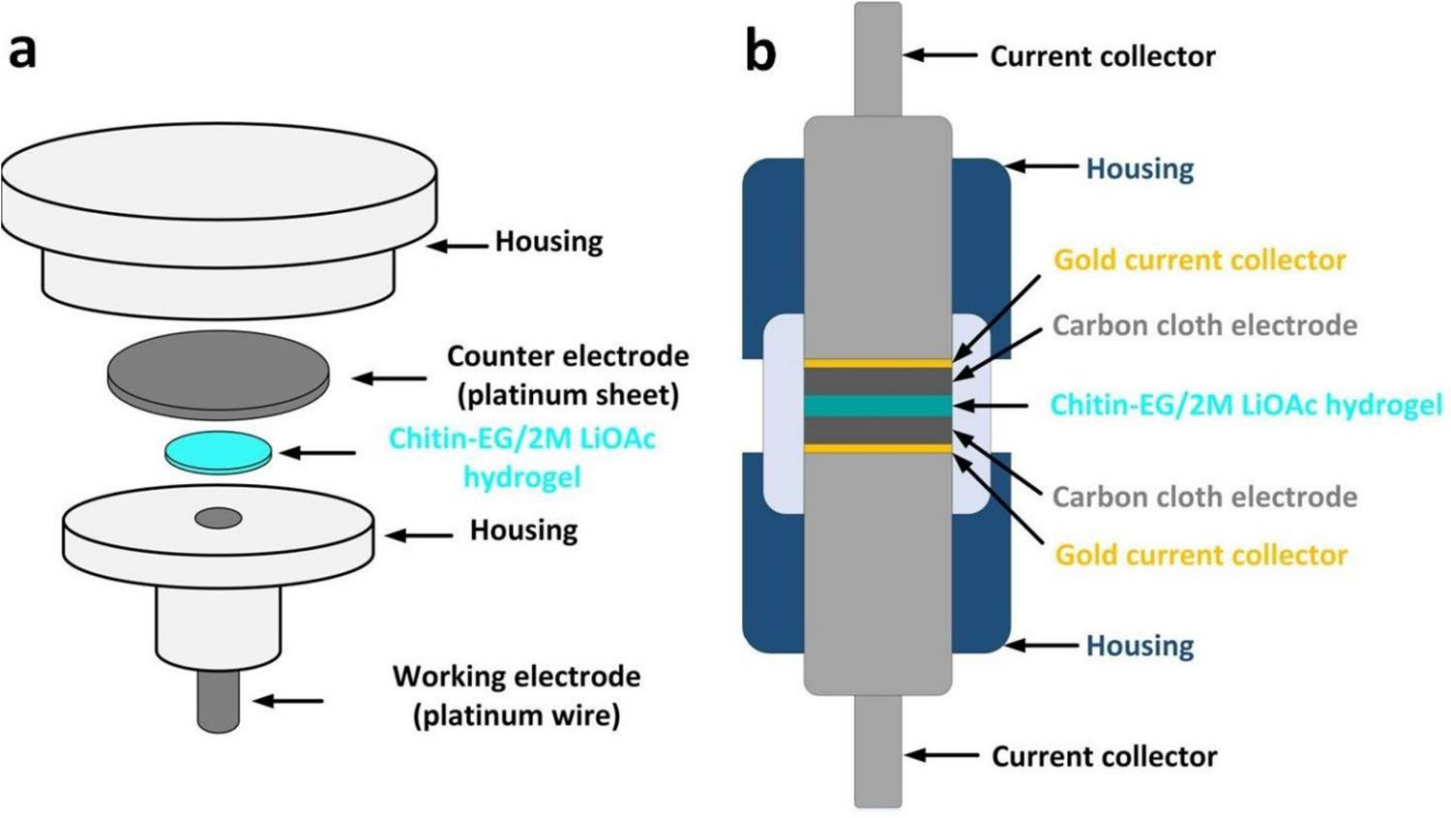
Schematic views of (**a**) the ionic conductivity measuring system; (**b**) cross-section of the electric double-layer capacitor (EDLC) assembled using the Swagelok^®^ system.

The ionic conductivity of the chitin–EG membrane in the form of a hydrogel was measured by electrochemical impedance spectroscopy (EIS). All measurements were performed at 25 °C in a frequency range from 100 kHz to 1 Hz with a potential amplitude of 10 mV. The investigated samples (13 mm discs) had previously been swelled for 24 h in a 2 M lithium acetate (LiOAc) aqueous solution, and were placed in a test vessel between two blocking electrodes made of platinum (Pt) (Fig. 1a). The ionic conductivity (*σ*) of the examined hydrogel electrolyte was calculated as:

2 $$ \sigma = \, t_{{\text{s}}} /(A \times R) $$

where *t*_s_ is the thickness of the swollen membrane, *A* is the surface area of the working electrode (0.0177 cm^2^), and *R* is the resistance of the membrane. For comparison, the ionic conductivity of a glass fiber membrane (Whatman GF/A) saturated in 2 M LiOAc aqueous solution was also evaluated.

### Assembly of electric double-layer capacitor (EDLC) cells

The electrochemical characteristics of the chitin–EG membrane as a hydrogel electrolyte were examined in an electric double-layer capacitor (EDLC) cell. The symmetric EDLC cell was assembled using a two-electrode Swagelok^®^ system (Fig. 1b). Activated carbon cloth (Kynol^®^ No ACC-507-20; 2000 m^2^ g^−1^) and gold discs (Au) were used in the test cell as electrode material and current collectors, respectively. The electrodes were shaped as 6 mm diameter discs, and the mass of a single electrode was in the range 4.6–4.8 mg. The chitin–EG membrane, after 24 h of treatment in a 2 M LiOAc aqueous solution, was applied as a hydrogel electrolyte. An EDLC cell assembled using the same electrode material and electrolyte as for the chitin–EG cell was used as a comparative system. In this EDLC, a glass fiber membrane (Whatman GF/A), with a pore diameter of 1.6 µm and a thickness of 0.3 mm, was used as the separator.

### Electrochemical measurements 

Electrochemical analysis of the EDLC cell with the chitin–EG hydrogel electrolyte was performed using potentiostatic EIS, cyclic voltammetry (CV), and galvanostatic charge/discharge (GCD) techniques. EIS was carried out using a μAutoLab FRA2 type III electrochemical system (EcoChemie, Netherlands) in a frequency range from 100 kHz to 0.01 Hz, with a sinusoidal excitation signal of amplitude 10 mV. CV measurements were performed using the same electrochemical system in the potential range 0–0.8 V and with different scan rates (2 to 100 mV s^−1^). Chronoamperometric measurements were performed at a constant current of 5 mA with the cell voltage stepped from 0 to 0.8 V, using an Atlas 0461 MBI multichannel electrochemical system (Atlas-Sollich, Poland). All electrochemical measurements were performed at room temperature (25 ºC).

### Assessment of membrane stability in 6 M KOH using FT-IR imaging

Samples of prepared membranes were immersed in 6 M KOH for 120 h. Assessment of chitin-EG membrane chemical stability was performed using a LUMOS II FT-IR microscope (Bruker). The material was tested in the ATR mode. The imaging was performed in an area of 1000 × 1000 µm. Stability was determined on the basis of the peak area which is characteristic of chitosan and occurs between 1700 and 1610 cm^−1^. 60 scans were collected for each spectrum. The results were processed using the OPUS software.

## Results and discussion

The selection of [BMIM][Ac] as a chitin solvent was motivated by the fact that this IL is one of the best-known representatives of this class of compounds, which contributes to its relatively low price and good market availability. [BMIM][Ac] is currently one of the best-studied ILs and has proved to be extremely effective in various applications, including the delignification of wood^44^, enhancement of the activity and stability of lipase^45^, CO_2_ capture^46^, and dissolution of cellulose^47^. Additionally, ionic liquids with a imidazolium ring showed a higher ability to dissolve chitin, and ionic liquid with a more basic anion such as acetate could exhibit better capability to break the strong hydrogen bond within chitin^48^. Additionally, while [Bmim][Cl] can only dissolve the non-crystal domains of a native chitin material, acetate anion led to dissolution of both non-crystal as well as the compact crystal domains of various chitin polymorphs^49^.

**Figure 2:**
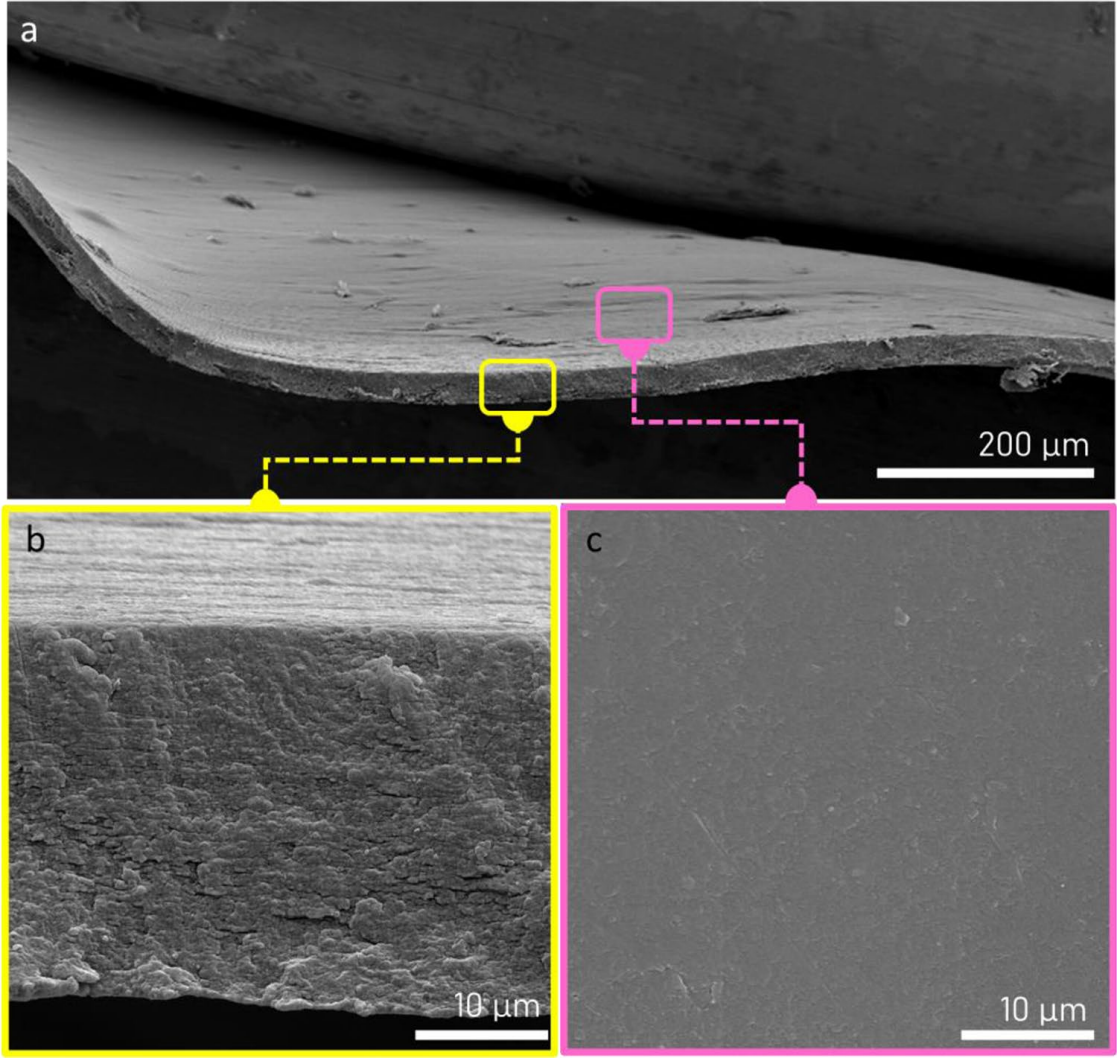
Scanning electron microscopy images showing (**a**) an overview, (**b**) the cross-section, and (**c**) the surface of prepared chitin–ethylene glycol thin films.

Scanning electron microscopy images of the chitin–EG films are shown in Fig. 2. The cross-section of the membrane (Fig. 2b) confirms the good homogeneity of the membrane obtained and shows that regenerated chains are oriented in parallel, forming a compact structure. The thickness of the membrane is 27 μm. It is visible that the surface of the obtained film is relatively smooth (Fig. 2c). This confirms that the presence of ethylene glycol did not cause discontinuities or porous structures when it was mixed with IL-regenerated chitin.

**Figure 3:**
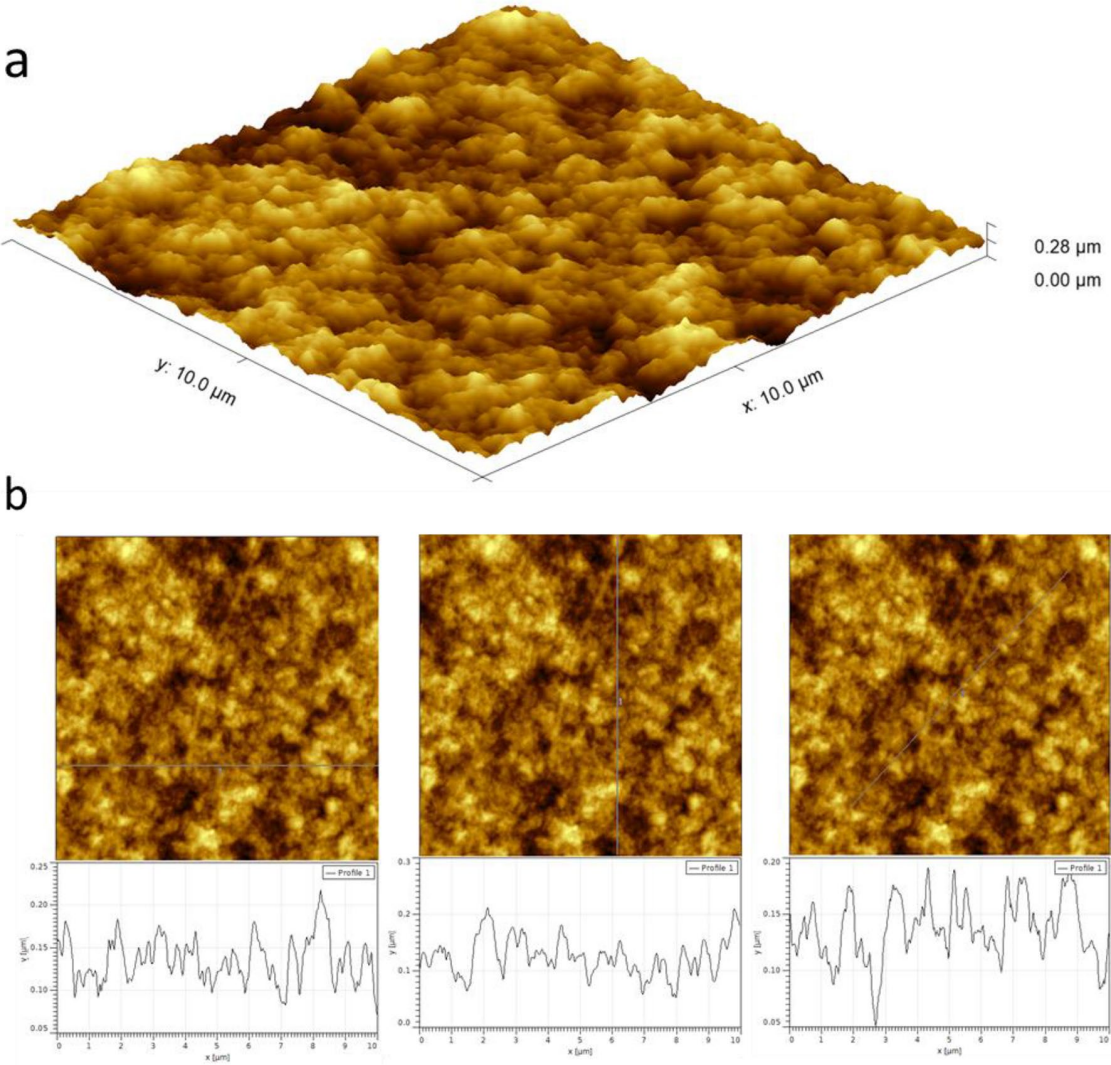
(**a**) AFM 3D surface topography, (**b**) AFM surface topography images with three different cross-sections measured. The size of the image is 10 × 10 μm.

**Figure 4:**
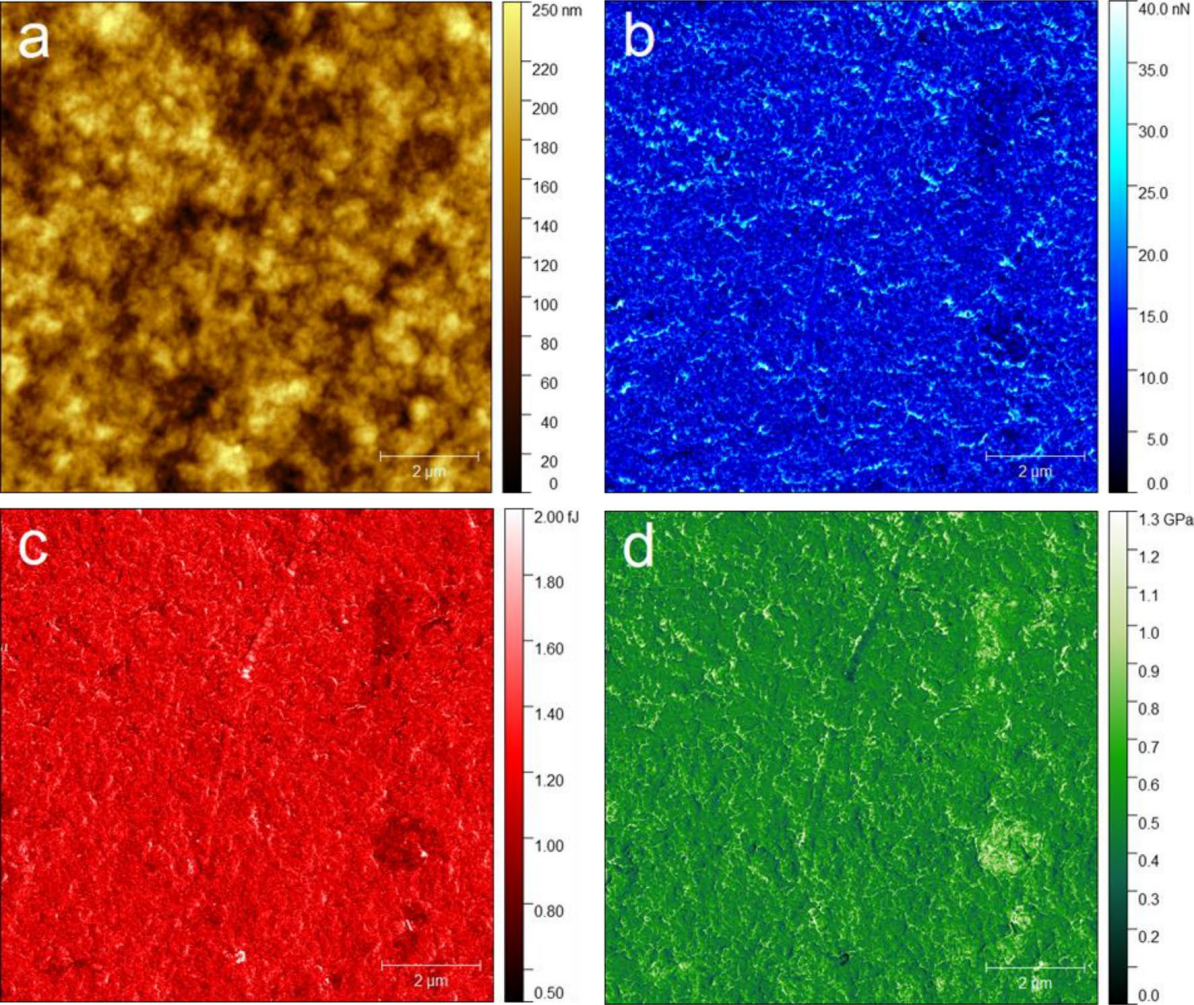
Comparison of different AFM images: (**a**) topography, (**b**) adhesion force, (**c**) energy dissipation, and (**d**) Young’s modulus. The image size is 10 × 10 μm.

Examination of the topography of the chitin–EG membrane shows a homogeneous and fine-grained surface, with an average height of 136.7 nm and a maximum height of 275.6 nm (Fig. 3). Measurement of 15 different grains revealed an average grain radius of 175 ± 45 nm. Mean roughness analysis gave an S_a_ value of 30.74 nm in the 10 × 10 μm image. There are no distinguished spots visible. Three nanomechanical properties were chosen for further investigation (Fig. 4): adhesion force, energy dissipation, and Young’s modulus. Investigation of adhesion reveals an even distribution of adhesion force across the image, with areas of slightly lower adhesion on the right side. These areas cover 4.2% of the overall image. The average measured adhesion force is 12.42 ± 0.26 nN. Multiple spots with adhesion around 25 nN are also visible; however, AFM adhesion images are always strongly dominated by topographical effects induced by the greater contact surface of the tip with the sample on the slopes of the surface. Similarly, as in the adhesion image, the analyses of energy dissipation and Young’s modulus show a regular distribution of values, except for two spots on the right side distinguished by lower energy dissipation and higher modulus. The mean measured values are 1.17 ± 0.01 fJ for energy dissipation and 0.54 ± 0.01 GPa for Young’s modulus. The spots visible on the right side of the image have Young’s modulus values of around 0.8 GPa. Analysis of the mechanical properties of obtained thin chitin-EG membranes indicate that they can be easily applied for construction of EDLC.

**Figure 5:**
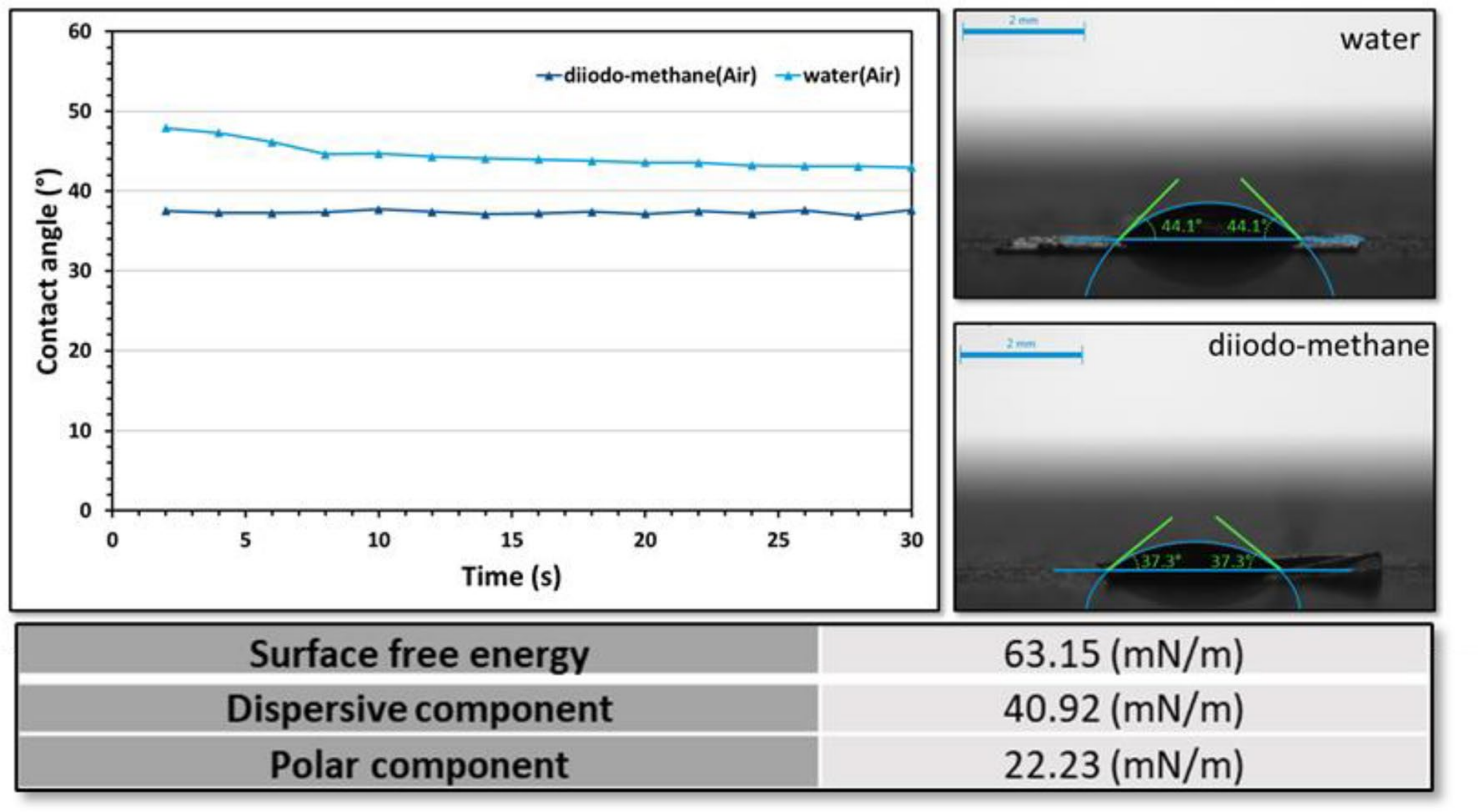
Water and diiodomethane contact angles and calculated surface free energy.

The contact angle is an essential parameter often used to assess the wettability of a solid surface by a liquid. It is defined as the angle, measured through the liquid phase, between the tangent of the gas–liquid interface at the intersection of gas, liquid, and solid, and the solid–liquid interface^50^. The contact angles of water (a polar liquid) and diiodomethane (a nonpolar liquid) as functions of the droplet interaction time are shown in Fig. 4. The initial contact angle of a water droplet on the surface of the chitin–EG membrane was 47°, decreasing slightly to a constant value of 43° in a few seconds as the water droplet interacted strongly with the membrane surface due to its hydrophilic character. The contact angle recorded for diiodomethane was constant and equal to 37.34° ± 0.22°. The final angle recorded for water is of more practical significance for chitin–EG, because liquid water is the medium in the environment of an EDLC. However, the obtained contact angle values indicate that chitin–EG thin film might be applied for both aqueous and non-aqueous electrolytes. This was confirmed by the swelling factor for chitin–EG with respect to a 2 M LiOAc electrolyte. The determined SF was 154 ± 3%, indicating the good swelling properties of the prepared gels^51^. The specific properties of a composite material depend strongly on its surface energy and specific interaction forces (e.g., hydrogen bonding, acid–base type interactions, dipole moments, charge transfer or electron acceptor–donor complexes)^52^. The calculated values of the total surface free energy and dispersive and polar components for chitin–EG thin films are presented in Fig. 5. Figure S4 demonstrates the images before and after impregnation with 2 M LiOAc electrolyte, which confirm that chitin-EG hydrogel suppress electrolyte leakage.

**Figure 6:**
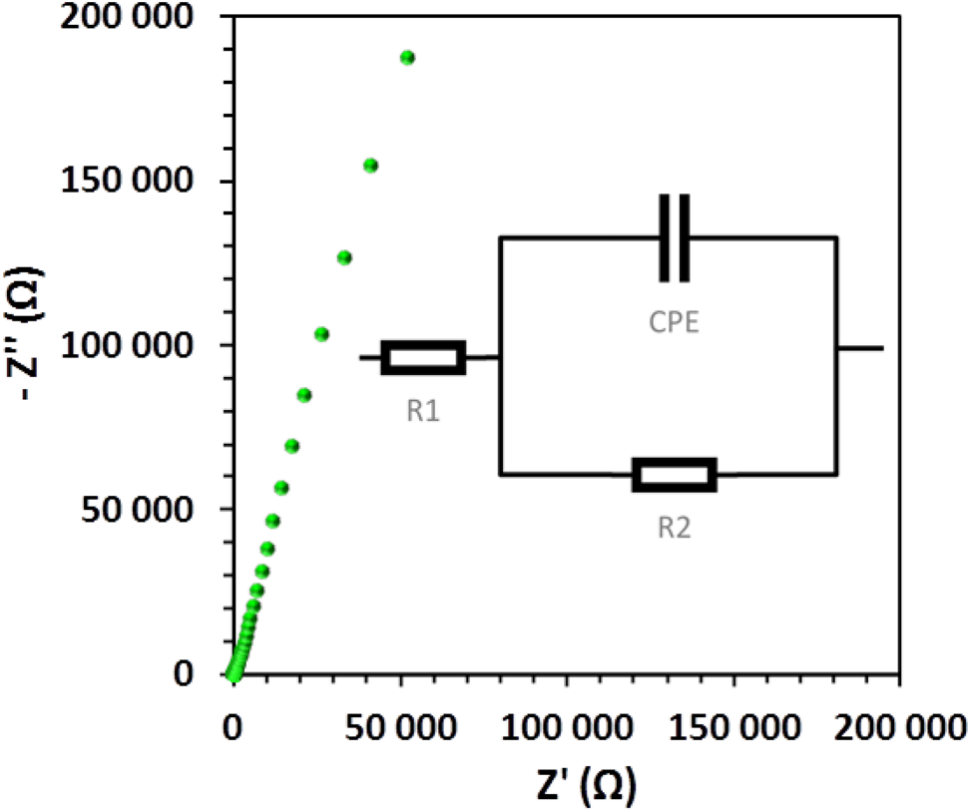
AC impedance spectra for chitin–EG hydrogel, with diagram of the fitting circuit.

Figure 6 shows a Nyquist plot for the chitin–EG hydrogel, accompanied by a diagram of the fitting circuit^53,54^. The values of ionic conductivity (*σ*), thickness in swollen state (*t*_*s*_), bulk resistance of electrolyte (*R*) and mass (*m*_*s*_) for the membranes examined in aqueous solution of 2 M LiOAc (24 h) are given in Table 1.

**Table 1:** Ionic conductivity (*σ*), bulk resistance of electrolyte (*R*), thickness in swollen state (*t*_*s*_), and mass (*m*_*s*_) for examined membranes in 2 M LiOAc aqueous solution (24 h) and equivalent circuit (EC) used in the EIS fitting method.

Membrane	*σ *(mS cm^−1^)	*R *(Ω)	*t*_*s*_ (cm)	*m*_*s*_ (g)	EC
Chitin–EG	15.7	23.7	0.0066	0.0115	R_1_(R_2_Q_1_)
Whatman GF/A 2 M	37.5	45.2	0.0300	0.0400	R_1_(R_2_Q_1_)
LiOAc	38.2	32.5	–	–	R_1_(R_2_Q_1_)

The ionic conductivity value of the chitin–EG hydrogel is 15.7 mS cm^−1^, while the value obtained for the Whatman GF/A separator under the same conditions was more than twice as high (37.5 mS cm^−1^). This phenomenon can be explained by differences in the spatial structure and the amount of absorbed electrolyte between the chitin–EG membrane and the Whatman GF/A separator. The chitin–EG hydrogel polymer chain network is tight and creates tunnels and gaps accessible to electrolyte ions simply by swelling, while the Whatman GF/A separator has pores with a predetermined diameter (easily accessible to electrolyte ions). However, the ionic conductivity of the tested chitin–EG hydrogel is comparable to that of other membranes of polysaccharide origin^19,55^.

**Figure 7:**
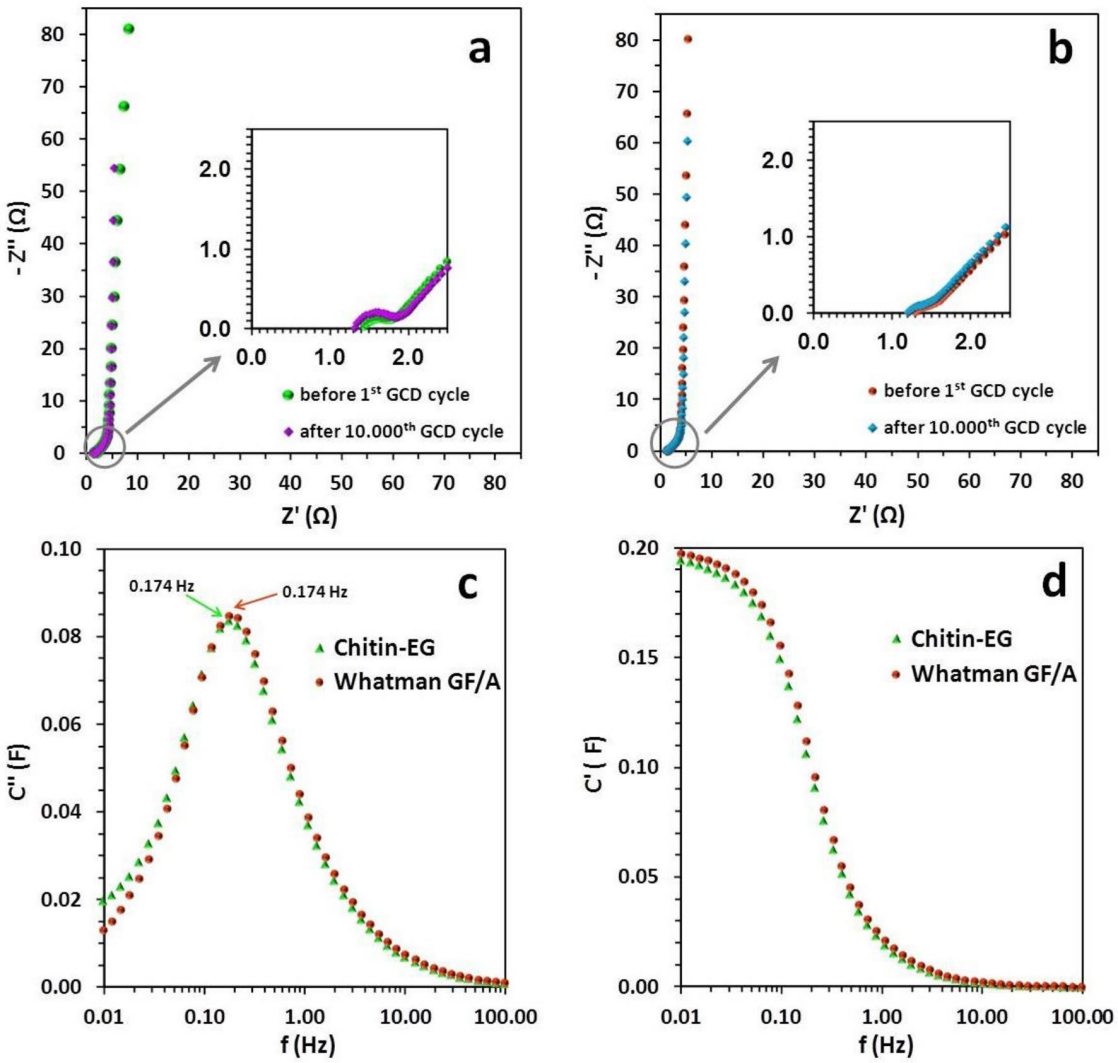
AC impedance spectra for capacitors with (**a**) chitin–EG hydrogel and (**b**) Whatman GF/A glass fiber separator. Evaluation of (**c**) the imaginary part of capacitance (C′′) with characteristic frequencies and (**d**) the real part of capacitance (C′) vs. frequency (on a logarithmic scale).

Nyquist plots for chitin–EG and Whatman GF/A test cells before the first and after the 10,000th GCD cycle are displayed in Fig. 7a,b. All obtained EIS spectra exhibit the typical shape for an EDLC. In the high-frequency range a relatively large semicircle is visible, and in the low-frequency range the EIS spectrum is a straight line parallel to the imaginary part of the impedance axis. Therefore, good capacitive behavior can be expected for the tested EDLC cells. Analyzing the insets of Fig. 7a,b (magnified high-frequency region) and literature data, the origin of the semicircular curve can be explained by three independent factors: (i) the interfacial resistances of the carbon material and (ii) the connection of the current collector and electroactive material, as well as (iii) the distribution of the pore structure and the electrolyte resistance therein. The two most important parameters extracted from EIS analysis and used to characterize the performance of EDLCs are the series resistance (*R*_*s*_) and the relaxation time (*t*_0_). The series resistance (R_s_) can be calculated straightforwardly by extrapolation of the high‐frequency part of the Nyquist plot to the condition Z′ = 0^56^. The two tested capacitors (chitin–EG and Whatman GF/A) had similar values of *R*_*s*_ before the GCD tests: 1.44 Ω and 1.31 Ω, respectively. The results correspond to the ionic conductivity measurements presented previously (Table 1) for swollen membranes. Despite the large gap between the ionic conductivity values of the swollen membranes, the difference in their *R*_*s*_ values is not as high as might be expected. This phenomenon can be attributed to the thickness of the membrane in the swollen state. Given that *R*_*s*_ is associated with the ionic resistance across the partition between the two facing electrodes, the limited swelling properties of the chitin–EG membrane may positively influence the bulk resistance of the hydrogel electrolyte. This resistance may be additionally boosted by the possible better inter-surface connection of chitin–EG hydrogel with the active material of the electrodes (obtained by pressing it into the carbon cloth fibers). This phenomenon is hardly observed in the case of an inductile glass fiber separator, and may influence ionic transportation between the electrodes. Moreover, detailed analysis of values of *R*_*s*_ after 10,000 GCD cycles revealed a slight drop to 1.33 Ω and 1.22 Ω for chitin–EG and Whatman GF/A EDLCs, respectively. These results correspond with a general trend (also confirmed by further CV and GCD measurements) for the electrochemical performance of the tested cells to improve with an increasing number of work cycles. This phenomenon is not directly related to the structure of the tested material or its pseudo-capacitive behavior (CV, GCD), but is rather caused by the enlargement of the active surface of the electrode material associated with gradual unblocking of the micropores filled with microbubbles of air.

Another crucial factor in the EIS analysis is the relaxation time constant (*t*_0_) resulting from detailed analysis of the dependence of the real and imaginary parts of capacitance (C′ and C′′) on the frequency. Figure 7c,d show the evaluation of the imaginary part of capacitance (C′) with characteristic frequencies and the real part of capacitance (C′) versus frequency. The relaxation time constants can be calculated using the following equation:

3 $$ t_{0} = { 1}/f_{0} $$

where *f*_0_ is the maximum of the C′′(ω) curve^20,57^. The peak frequencies of the C′′(ω) curves were determined and are presented on the graph (Fig. 7c). Relaxation time values obtained for the EDLC test cells exhibit a trend similar to the *R*_*s*_ values calculated from EIS measurements. Both EDLC cells had the same time constant of 5.8 s. The relaxation time defines the boundary between capacitive behavior and resistive behavior of an EDLC; therefore, it can be assumed that a capacitor with a chitin–EG hydrogel electrolyte will exhibit similar values of specific capacitance as the reference cell.

**Figure 8:**
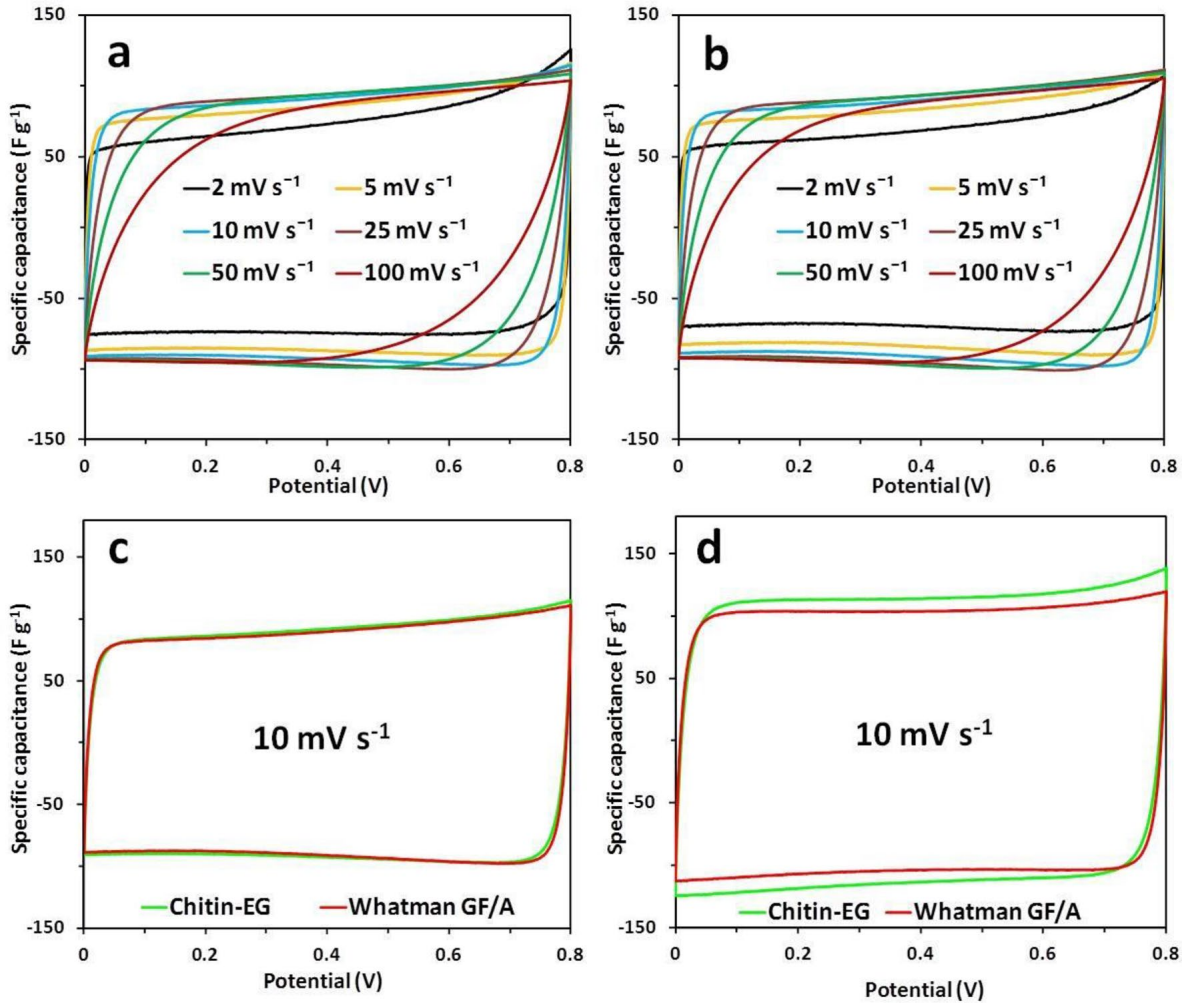
CV curves of EDLC test cells with (**a**) chitin–EG hydrogel and (**b**) Whatman GF/A glass fiber separator at different scan rates. Comparison of voltammograms recorded at the scan rate 10 mV s^−1^ (**c**) before the GCD test and (**d**) after 10,000 cycles of GCD.

Figure 8a,b show cyclic voltammograms at different scan rates (from 2 to 100 mV s^−1^) recorded for the chitin–EG capacitor and for the Whatman GF/A reference cell. The shape of the CV curves for both tested EDLC cells can be described as nearly rectangular, indicating good charge propagation (up to 10 mV s^−1^). The voltammograms show no significant deviations (such as redox peaks) in the full spectrum of scan rates used, which may be interpreted as a lack of faradaic capacitance within the examined EDLC cells. Thus, the assembled test cells should be treated in further calculations as regular EDLC devices^58^. The CV curves in Fig. 8a,b recorded at higher scan rates (from 25 mV s^−1^ inclusive) indicate a significant drop in charge propagation; nevertheless, this is a standard phenomenon for EDLCs at higher scan rates^59^. The voltammograms in Fig. 8c,d were recorded at a scan rate of 10 mV s^−1^ for both tested EDLCs: before the galvanostatic charge/discharge test (GCD) (Fig. 8c) and after 10,000 cycles of GCD (Fig. 8d). The comparison in Fig. 8c shows that before the GCD test, the EDLC cell with chitin–EG hydrogel electrolyte and the reference EDLC cell with Whatman GF/A separator had similar CV characteristics (72 F g^−1^ and 71 F g^−1^, respectively). The specific capacitance values for all capacitor cells were calculated using the following equation:

4 $$ C_{sp} = \smallint I{\text{d}}t/\left( {{\text{d}}E/{\text{d}}t} \right)m^{{ - {1}}} $$

where *I* is the current, d*E*/d*t* is the potential scan rate, and *m* is the total mass of the active electrode material. An interesting phenomenon is observed in Fig. 8d, which shows the CV curves (10 mV s^−1^) after 10,000 GCD cycles. Here, the specific capacitance values calculated for the chitin–EG and Whatman GF/A EDLCs are 88 F g^−1^ and 82 F g^−1^, respectively. This difference indicates that the test EDLC cell with the chitin–EG hydrogel may exhibit high cyclic repeatability (better than that of the reference cell). Moreover, the absence of any deviations within the chitin–EG CV curve proves that even after 10,000 cycles of charging and discharging, the hydrogel material used was electrochemically stable and did not decompose.

**Figure 9:**
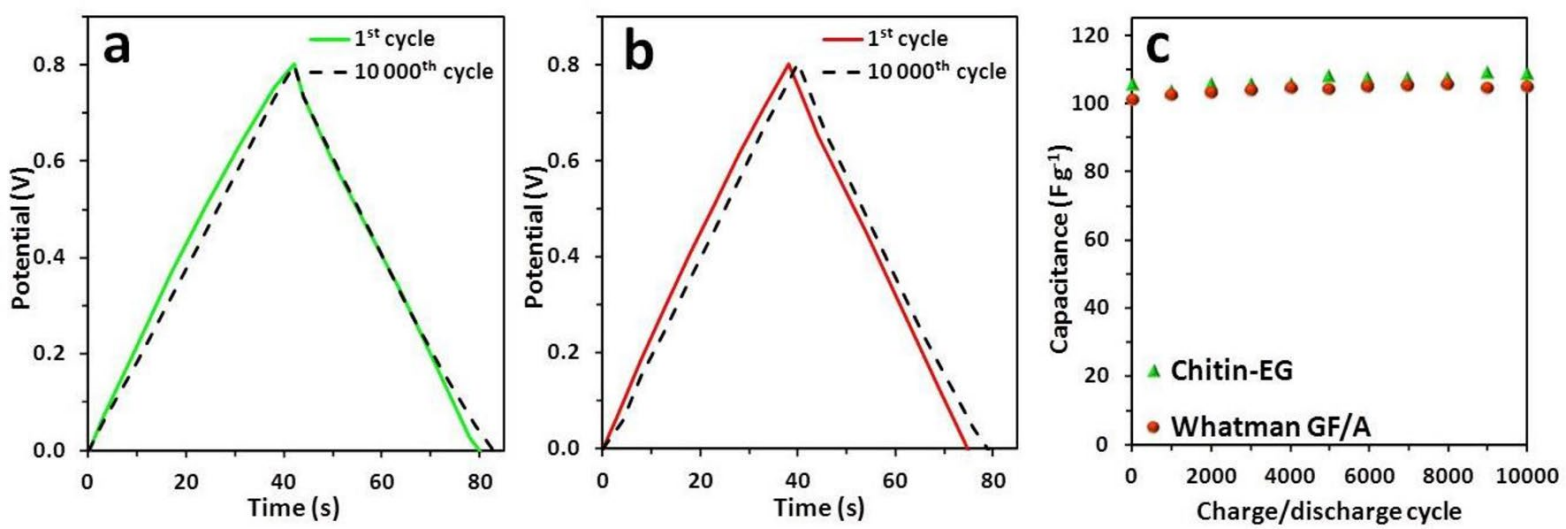
Galvanostatic charge/discharge curves at the 1st and 10,000th cycle for EDLC test cells with (**a**) chitin–EG hydrogel and (**b**) Whatman GF/A glass fiber separator. (**c**) Cyclic stability over 10,000 cycles for tested EDLC cells.

To test the cycling stability of the chitin–EG and Whatman GF/A test cells, galvanostatic charge/discharge (GCD) tests were performed. Figure 9a,b show the GCD profiles recorded for tested EDLC cells at the 1st and 10,000th cycle (in a potential range from 0 to 0.8 V). Figure 9a,b show that the potential/time dependency curves for the chitin–EG and Whatman GF/A test cells exhibit a triangular shape for both the 1st and 10,000th cycles. This is a typical GCD profile for EDLCs and indicates the non-faradaic nature of the capacitive properties (proved also by cyclic voltammetry tests) and good cyclic repeatability^60^. Moreover, detailed analysis of Fig. 9b shows that the 10,000th GCD profile recorded for the Whatman GF/A EDLC cell exhibits a slight shift of the curve relative to the initial one.

This result indicates that an EDLC cell with chitin–EG hydrogel has better cyclic repeatability than the reference cell, which may be linked to a better inter-surface connection of the chitin–EG hydrogel with the active material of the electrodes (noted in the EIS analysis). Specific capacitance values were obtained from the corresponding GCD curves according to the equation:

5 $$ C_{{{\text{sp}}}} = \, I/\left( {{\text{d}}U/{\text{d}}t} \right)m^{{ - {1}}} $$

where *I* is the discharge current, d*U*/d*t* is the slope of the discharge curve, and *m* is the total mass of the active electrode material. Table 2 gives specific capacitance values obtained after the 1st and 10,000th GCD cycle with a constant current of 5 mA.

**Table 2:** Specific capacitance (*C*_sp_) of EDLC cells, calculated from the GCD test after 1st and 10,000th cycle.

EDLC	*C*_sp_ (F g^−1^)	
	1st cycle	10,000th cycle
Chitin–EG	106	109
Whatman GF/A	101	105

The chitin–EG EDLC cell has a higher specific capacitance value at both the 1st (106 F g^−1^) and 10,000th (109 F g^−1^) cycle, as well as excellent repeatability. The Whatman GF/A EDLC cell has slightly lower but comparable specific capacitance values (101 F g^−1^ at the 1st cycle and 105 F g^−1^ at the 10,000th cycle of GCD). Analysis of other EDLCs with comparable architecture described in the literature (Table 3) shows that the electrochemical characteristic of the chitin–EG capacitor is competitive with other cells containing polysaccharide-based hydrogel electrolytes (e.g. chitin composites, cellulose or chitosan). Despite the low ionic conductivity of the chitin–EG hydrogel electrolyte, the tested EDLC exhibits a specific capacitance value comparable with those of other materials. A similar phenomenon can be observed for cross-linked chitosan membranes, which indicates that swelling properties of the hydrogel matrix have a large impact on the EDLC’s electrochemical performance. Thus, crosslinking in the case of chitosan, and preservation of the natural unmodified crystalline structure in the case of chitin, may play crucial roles in possible electrochemical applications of these polysaccharides. Figure 9c shows the cycling stability of the GCD profiles of the tested EDLC cells. The EDLC with the chitin–EG hydrogel exhibited excellent cycle life, without significant drops in capacitance per 10,000 cycles. These results correspond to the values obtained by cyclic voltammetry and indicate that, despite the lower ionic conductivity, the chitin–EG hydrogel can be recognized as a fully functional EDLC component. Moreover, chitin–EG hydrogel in an EDLC exhibits superior capacitance retention over 10,000 GCD cycles and may be competitive with the commercial glass fiber separator. Figure 9c shows further that specific capacitance increases after 10,000 GCD cycles; this phenomenon should be investigated in detail in future research. However, since this growth is proportional and can be observed for both the chitin–EG EDLC and the Whatman GF/A reference cell, it may be associated with other test cell components (e.g., current collectors or electrode material). Most likely, it is caused by increasing penetration of the electrode pores and specific surface activation by electrolyte ions that are pushed through the electrode bulk.

**Table 3:** Ionic conductivity (*σ*) and specific capacitance (*C*_sp_) values for EDLC cells with tested membranes, compared with literature data.

Matrix	Liquid phase	Electrode material	*σ *(mS cm^−1)^	Voltage range (V)	*C*_sp_ (F g^−1^) (from GCD)	Ref
Chitin–EG	2 M LiOAc/H_2_O	ACC	15.7	0–0.8	109	This work
Chitin	1 M Li_2_SO_4_/H_2_O	ACC	66	0–0.8	96	^17^
Chitin/cellulose	1 M Li_2_SO_4_/H_2_O	ACC	50	0–0.8	95	^17^
Carboxymethylchitin	[BMIm][Ac]	–	0.6	–	–	^23^
Carboxymethylchitin	[BMIm][Ac]	–	1.2	–	–	^24^
Chitin/chitosan	1 M LiOAc/H_2_O	ACC	–	0–0.8	96	^20^
Cellulose	2 M LiOAc/H_2_O	ACC	38.8	0–0.8	22	^16^
Chitosan	2 M LiOAc/H_2_O	ACC	39.7	0–0.8	107	^19^
Chitosan/NaOH	2 M LiOAc/H_2_O	ACC	14.3	0–0.8	106	^19^
Chitosan/glutaraldehyde	2 M LiOAc/H_2_O	ACC	13.0	0–0.8	106	^19^
Chitosan	1 M LiOAc/H_2_O	ACC	–	0–0.8	87	^20^
Whatman GF/A	2 M LiOAc/H_2_O	ACC	37.5	0–0.8	105	This work
Whatman GF/A	1 M Li_2_SO_4_/H_2_O	ACC	54	0–0.8	98	^17^
Whatman GF/A	2 M LiOAc/H_2_O	ACC	38.1	0–0.8	106	^19^

**Figure 10:**
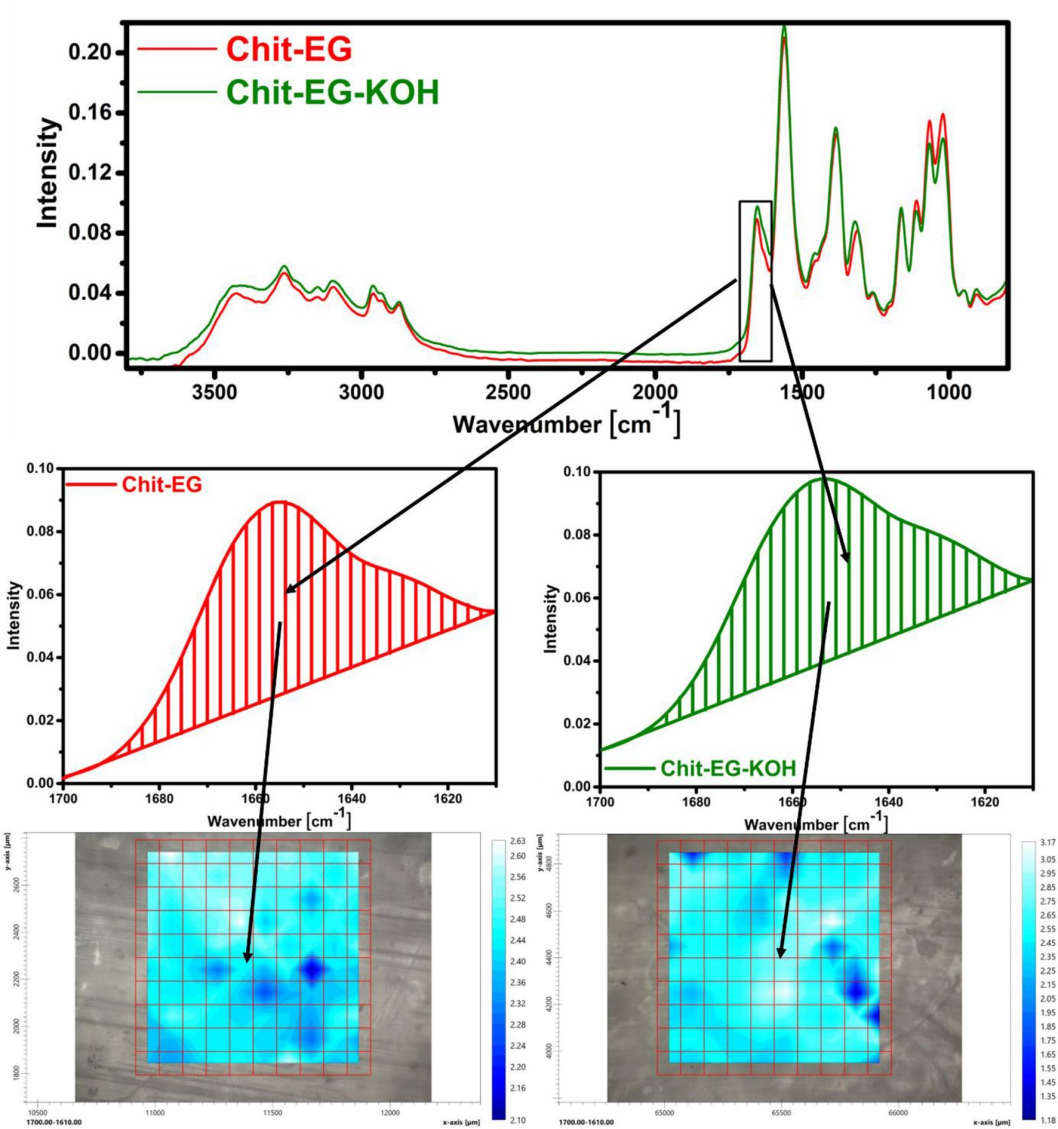
FTIR microscopic analysis of the Chit-EG membrane as prepared (red) and after ageing for 5 days in 6 M KOH (green).

The chemical stability of pristine chitin-EG membrane and corresponding membrane immersed in 6 M KOH for 120 h, was tested by FTIR imaging microscopic analysis (Fig. 10). The registered two spectra and images appear very similar. Consequently, we can easily conclude that the chitin-EG membrane has good stability in alkaline environment, at least in the limits of the ageing protocol we employed.

## Conclusions

Chitin–ethylene glycol membranes were prepared effectively using an ionic liquid (1-butyl-3-methylimidazolium acetate) as the chitin solvent. Structural examination revealed the smooth and homogeneous morphology of the obtained membranes and confirmed that mixing with ethylene glycol does not cause the formation of discontinuous or porous structures in the cross-section of the membrane. The tested EDLC cell with a chitin–EG hydrogel exhibits good electrochemical performance and specific capacitance, superior to those of the Whatman GF/A glass fiber separator reference cell. The remarkable mechanical and electrochemical properties of the prepared chitin-based membranes and hydrogel electrolytes, coupled with their simple manufacturing process, make them suitable for use as renewable alternatives to synthetic polymers, which may help to reduce the environmental impact associated with (i) seafood waste and (ii) traditional GPEs and their manufacturing processes. The prepared hydrogel electrolyte appears to be a promising component for the development of green electrochemical capacitors.

## Supplementary Information

Supplementary Figures.

## Data Availability

All data generated or analysed during this study are included in this published article and its supplementary information files.

## References

[1] Yan N, Chen X (2015). Don’t waste seafood waste. Nature.

[2] Bastiaens L, Soetemans L, D’Hondt E, Elst K (2019). Sources of chitin and chitosan and their Isolation. Chitin Chitosan.

[3] Yadav M (2019). Seafood waste: A source for preparation of commercially employable chitin/chitosan materials. Bioresour. Bioprocess..

[4] Tsurkan MV (2021). Progress in chitin analytics. Carbohyd. Polymers.

[5] Suryawanshi N, Eswari JS (2021). Chitin from seafood waste: Particle swarm optimization and neural network study for the improved chitinase production. J. Chem. Technol. Biotechnol..

[6] Tharanathan, R. N. & Kittur, F. S. Chitin—The undisputed biomolecule of great potential. **43**, 61–87. 10.1080/10408690390826455 (2010). 10.1080/10408690390826455 12587986

[7] Naghdi T (2020). Chitin nanofiber paper toward optical (bio)sensing applications. ACS Appl. Mater. Interfaces..

[8] Fic K, Platek A, Piwek J, Frackowiak E (2018). Sustainable materials for electrochemical capacitors. Mater. Today.

[9] Okhawilai M, Pattananuwat P (2021). Sustainable electroactive materials for energy storage. Curr. Opin. Green Sustain. Chem..

[10] Islam MA (2021). Biomass-derived cellulose nanofibrils membrane from rice straw as sustainable separator for high performance supercapacitor. Ind. Crops Prod..

[11] Peter S (2020). Chitin and chitosan based composites for energy and environmental applications: A review. Waste Biomass Valoriz..

[12] Yamagata M, Soeda K, Ikebe S, Yamazaki S, Ishikawa M (2013). Chitosan-based gel electrolyte containing an ionic liquid for high-performance nonaqueous supercapacitors. Electrochim. Acta.

[13] Zhong C (2015). A review of electrolyte materials and compositions for electrochemical supercapacitors. Chem. Soc. Rev..

[14] Huang Y (2017). An intrinsically stretchable and compressible supercapacitor containing a polyacrylamide hydrogel electrolyte. Angew. Chem. Int. Ed..

[15] Torres FG, De-la-Torre GE (2021). Algal-based polysaccharides as polymer electrolytes in modern electrochemical energy conversion and storage systems: A review. Carbohyd. Polymer Technol. Appl..

[16] Kasprzak D, Stępniak I, Galiński M (2018). Electrodes and hydrogel electrolytes based on cellulose: Fabrication and characterization as EDLC components. J. Solid State Electrochem..

[17] Kasprzak D, Galiński M (2021). Chitin and chitin-cellulose composite hydrogels prepared by ionic liquid-based process as the novel electrolytes for electrochemical capacitors. J. Solid State Electrochem..

[18] Choudhury NA, Northrop PWC, Crothers AC, Jain S, Subramanian VR (2012). Chitosan hydrogel-based electrode binder and electrolyte membrane for EDLCs: Experimental studies and model validation. J. Appl. Electrochem..

[19] Nowacki K, Galiński M, Stępniak I (2019). Synthesis and characterization of modified chitosan membranes for applications in electrochemical capacitor. Electrochim. Acta.

[20] Stepniak I (2016). A novel chitosan/sponge chitin origin material as a membrane for supercapacitors—Preparation and characterization. RSC Adv..

[21] Rolandi M, Rolandi R (2014). Self-assembled chitin nanofibers and applications. Adv. Coll. Interface. Sci..

[22] Kameda T, Miyazawa M, Ono H, Yoshida M (2005). Hydrogen bonding structure and stability of α-chitin studied by 13C solid-state NMR. Macromol. Biosci..

[23] Latifi M, Ahmad A, Hassan NH, Ben Youcef H, Kaddami H (2021). Towards the application of carboxymethyl chitin/ionic liquid blend as polymer electrolyte membrane for aqueous batteries. Carbohyd. Polym..

[24] Latifi M (2020). Chemical modification and processing of chitin for sustainable production of biobased electrolytes. Polymers.

[25] Satam CC, Meredith JC (2021). Increasing efficiency of the homogenization process for production of chitin nanofibers for barrier film applications. Carbohyd. Polym..

[26] Martin-Martinez FJ, Jin K, Barreiro DL, Buehler MJ (2018). The rise of hierarchical nanostructured materials from renewable sources: Learning from nature. ACS Nano.

[27] Zhu S, Tang Y, Lin C, Liu XY, Lin Y (2021). Recent advances in patterning natural polymers: From nanofabrication techniques to applications. Small Methods.

[28] Yuan Y, Hong S, Lian H, Zhang K, Liimatainen H (2020). Comparison of acidic deep eutectic solvents in production of chitin nanocrystals. Carbohyd. Polym..

[29] Guo X (2019). Electroassembly of chitin nanoparticles to construct freestanding hydrogels and high porous aerogels for wound healing. ACS Appl. Mater. Interfaces..

[30] Borić M, Puliyalil H, Novak U, Likozar B (2018). An intensified atmospheric plasma-based process for the isolation of the chitin biopolymer from waste crustacean biomass. Green Chem..

[31] Vasilieva T (2017). Formation of low molecular weight oligomers from chitin and chitosan stimulated by plasma-assisted processes. Carbohyd. Polym..

[32] Liu W (2020). Liquid crystalline and rheological properties of chitin whiskers with different chemical structures and chargeability. Int. J. Biol. Macromol..

[33] Isogai A (2022). TEMPO-catalyzed oxidation of polysaccharides. Polym. J..

[34] Liu P (2021). Unexpected selective alkaline periodate oxidation of chitin for the isolation of chitin nanocrystals. Green Chem..

[35] Huang J, Zhong Y, Wei P, Cai J (2021). Rapid dissolution of β-chitin and hierarchical self-assembly of chitin chains in aqueous KOH/urea solution. Green Chem..

[36] Shamshina JL, Berton P, Rogers RD (2019). Advances in functional chitin materials: A review. ACS Sustain. Chem. Eng..

[37] Shamshina JL (2019). Chitin in ionic liquids: Historical insights into the polymer’s dissolution and isolation. A review. Green Chem..

[38] Jaworska MM (2018). Modification of chitin structure with tailored ionic liquids. Carbohyd. Polym..

[39] Silva SS, Mano JF, Reis RL (2017). Ionic liquids in the processing and chemical modification of chitin and chitosan for biomedical applications. Green Chem..

[40] Barber PS, Kelley SP, Griggs CS, Wallace S, Rogers RD (2014). Surface modification of ionic liquid-spun chitin fibers for the extraction of uranium from seawater: Seeking the strength of chitin and the chemical functionality of chitosan. Green Chem..

[41] Tripathi AK (2021). Ionic liquid-based solid electrolytes (ionogels) for application in rechargeable lithium battery. Mater. Today Energy.

[42] King C (2017). A platform for more sustainable chitin films from an ionic liquid process. Green Chem..

[43] Sievers J (2020). Determination of hydrogel swelling factors by two established and a novel non-contact continuous method. J. Appl. Polymer Sci..

[44] Sun N (2009). Complete dissolution and partial delignification of wood in the ionic liquid 1-ethyl-3-methylimidazolium acetate. Green Chem..

[45] Kaar JL, Jesionowski AM, Berberich JA, Moulton R, Russell AJ (2003). Impact of ionic liquid physical properties on lipase activity and stability. J. Am. Chem. Soc..

[46] Shiflett MB, Drew DW, Cantini RA, Yokozeki A (2010). Carbon dioxide capture using ionic liquid 1-butyl-3-methylimidazolium acetate. Energy Fuels.

[47] Andanson JM (2014). Understanding the role of co-solvents in the dissolution of cellulose in ionic liquids. Green Chem..

[48] Deng L, Zhang LM (2020). Rheological characteristics of chitin/ionic liquid gels and electrochemical properties of regenerated chitin hydrogels. Colloids Surf. A.

[49] Wu Y, Sasaki T, Irie S, Sakurai K (2008). A novel biomass-ionic liquid platform for the utilization of native chitin. Polymer.

[50] Wang J, Wu Y, Cao Y, Li G, Liao Y (2020). Influence of surface roughness on contact angle hysteresis and spreading work. Colloid Polym. Sci..

[51] Liao J, Hou B, Huang H (2022). Preparation, properties and drug controlled release of chitin-based hydrogels: An updated review. Carbohyd. Polym..

[52] Shi B, Zhao S, Jia L, Wang L (2007). Surface characterization of chitin by inverse gas chromatography. Carbohyd. Polym..

[53] Gupta H (2017). Effect of temperature on electrochemical performance of ionic liquid based polymer electrolyte with Li/LiFePO4 electrodes. Solid State Ionics.

[54] Tripathi AK (2017). Quasi solid-state electrolytes based on ionic liquid (IL) and ordered mesoporous matrix MCM-41 for supercapacitor application. J. Solid State Electrochem..

[55] Shukur MF, Ithnin R, Kadir MFZ (2014). Electrical characterization of corn starch-LiOAc electrolytes and application in electrochemical double layer capacitor. Electrochim. Acta.

[56] Tõnurist K, Thomberg T, Jänes A, Kink I, Lust E (2012). Specific performance of electrical double layer capacitors based on different separator materials in room temperature ionic liquid. Electrochem. Commun..

[57] Taberna PL, Simon P, Fauvarque JF (2003). Electrochemical characteristics and impedance spectroscopy studies of carbon-carbon supercapacitors. J. Electrochem. Soc..

[58] Fic K, Lota G, Meller M, Frackowiak E (2012). Novel insight into neutral medium as electrolyte for high-voltage supercapacitors. Energy Environ. Sci..

[59] Huo P (2019). Crosslinked quaternized poly(arylene ether sulfone) copolymer membrane applied in an electric double-layer capacitor for high energy density. J. Appl. Polym. Sci..

[60] Pandey GP, Hashmi SA, Kumar Y (2010). Performance studies of activated charcoal based electrical double layer capacitors with ionic liquid gel polymer electrolytes. Energy Fuels.

