# Supplementary material for: Ionic liquid-assisted synthesis of chitin–ethylene glycol hydrogels as electrolyte membranes for sustainable electrochemical capacitors
Source: Sci Rep. 2022 May 25;12:8861. doi: 10.1038/s41598-022-12931-w (PMC9132938; doi:10.1038/s41598-022-12931-w)

***SUPPLEMENTARY INFORMATION***

***Figure S1****. FT-IR spectrum of 1-butyl-3-methylimidazolium Acetate [Bmim][Ac]*

IR υ_max_ [cm^-1^] = 2964, 2932, 2870, 1566, 1461, 1420, 1381, 1333, 1169, 1107, 1001, 945, 900, 747, 692, 652.


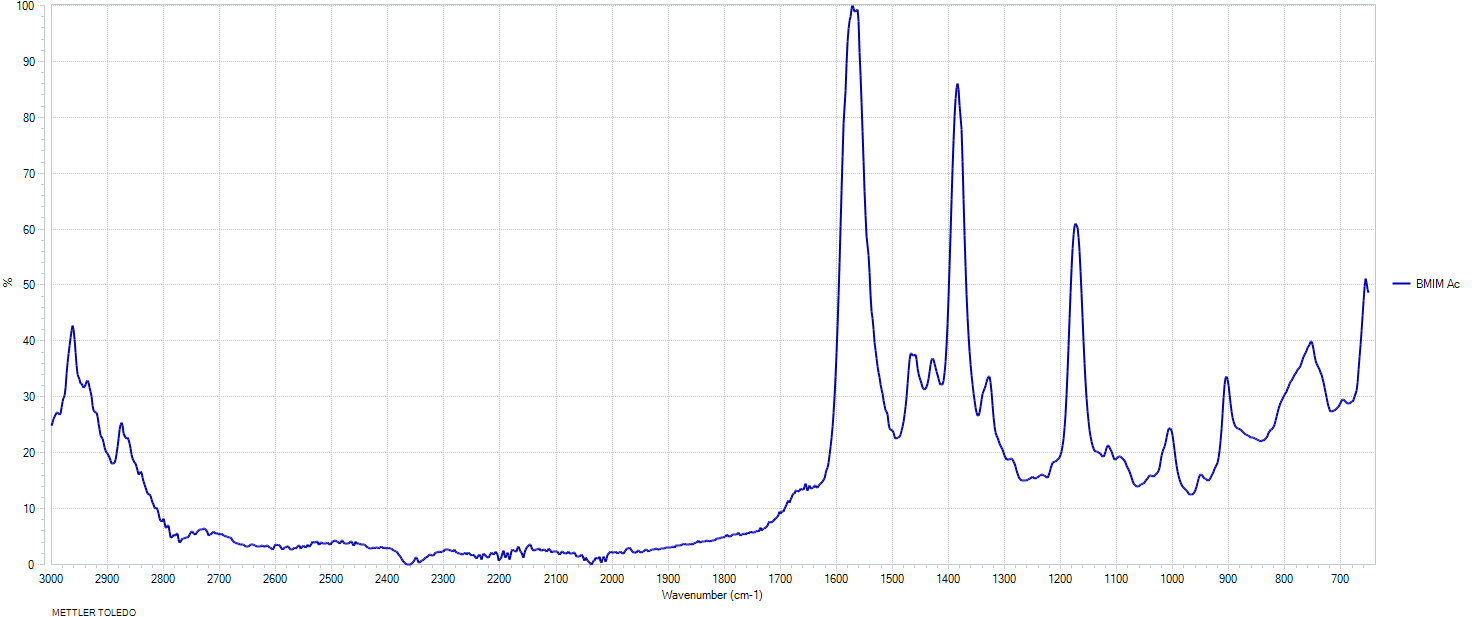


***Figure S2****. ^1^H NMR spectrum of 1-butyl-3-methylimidazolium Acetate [Bmim][Ac]*

^1^H NMR (400 MHz, DMSO-*d_6_*) δ [ppm] = 0.85 (3H, t, *J* = 7.4 Hz, -CH_2_-CH_3_); 1.21 (2H, sext, *J* = 7.4 Hz, -CH_2_-CH_3_); 1.57 (3H, s, OCO-CH_3_); 1.73 (2H, qu, *J* = 7.3 Hz, CH_2_-CH_2_-CH_2_); 3.87 (3H, s, N-CH_3_); 4.18 (2H, t, *J* = 7.2 Hz, N-CH_2_-CH_2_); 7.82 (1H, s, CH_3_-N=CH-CH); 7.90 (1H, s, CH_3_-N=CH-CH); 9.96 (1H, s, N-CH=N)


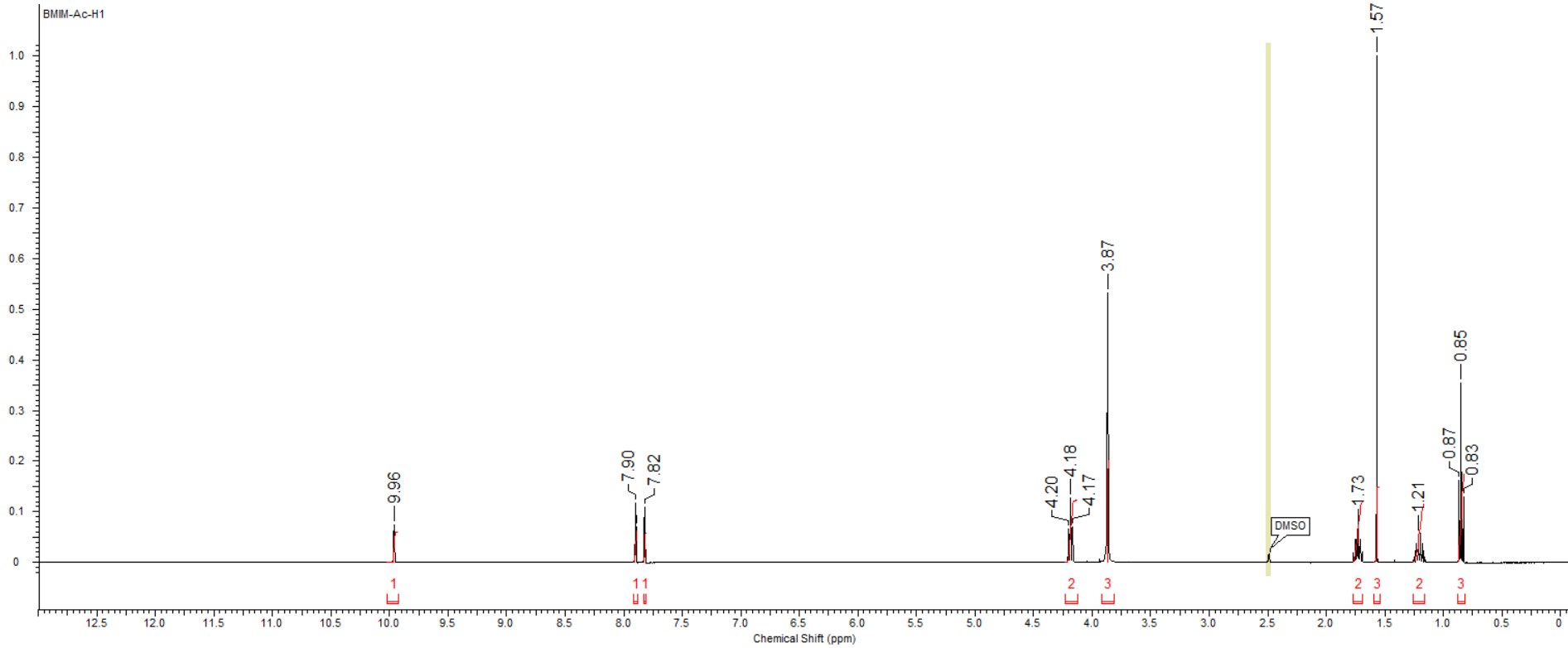


***Figure S3****. ^13^C NMR spectrum of 1-butyl-3-methylimidazolium Acetate [Bmim][Ac]*

***
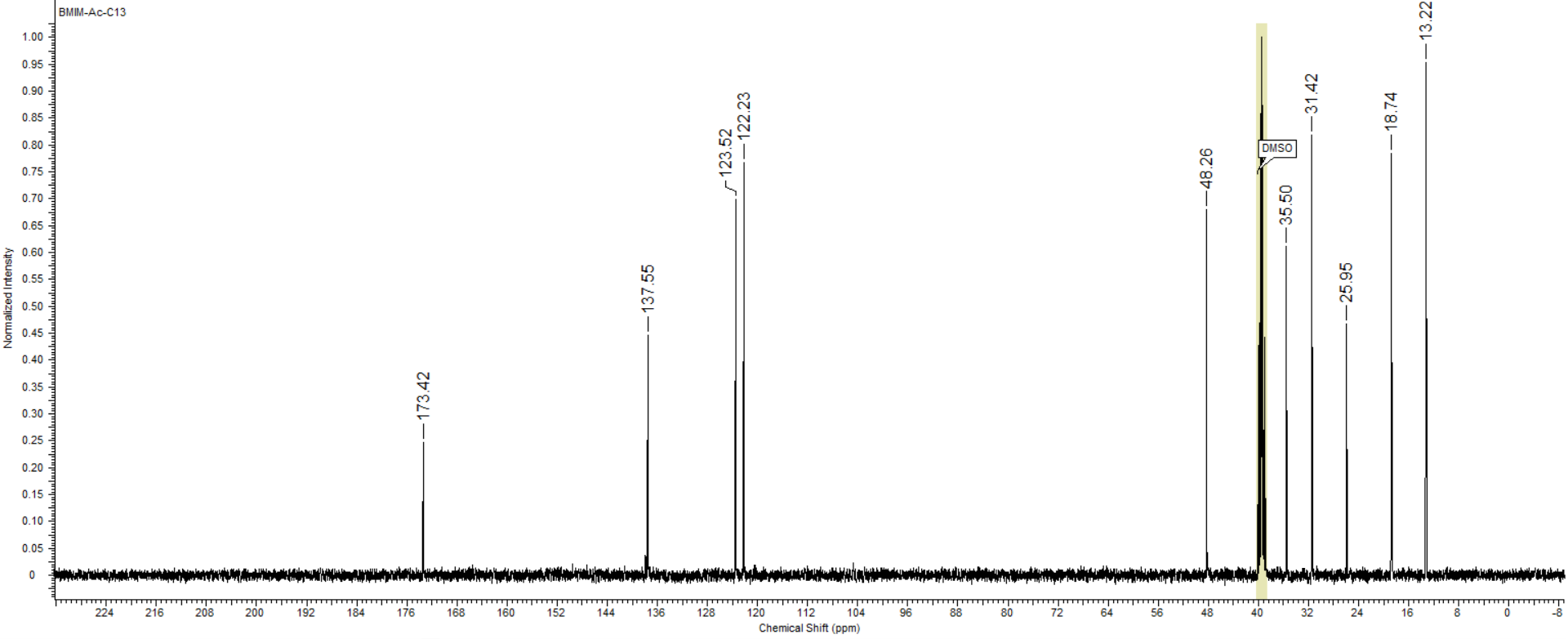
***

^13^C NMR (100 MHz, DMSO*-d_6_*) δ [ppm] = 13.2 (-CH_2_-CH_3_), 18.7 (-CH_2_-CH_3_), 26.0 (OCO-CH_3_), 31.4 (CH_2_-CH_2_-CH_2_), 35.5 (N-CH_3_), 48.3 (N-CH_2_-CH_2_), 122.2 (CH_3_-N=CH-CH), 123.5 (CH_3_-N=CH-CH), 137.6 (N-CH=N), 173.4 (OCO-CH_3_).

***Figure S4****. Optical microscopy images of (a) prepared dry chitin-EG membrane, and (b) chitin-EG membrane swollen with 2M Lithium acetate electrolyte.*


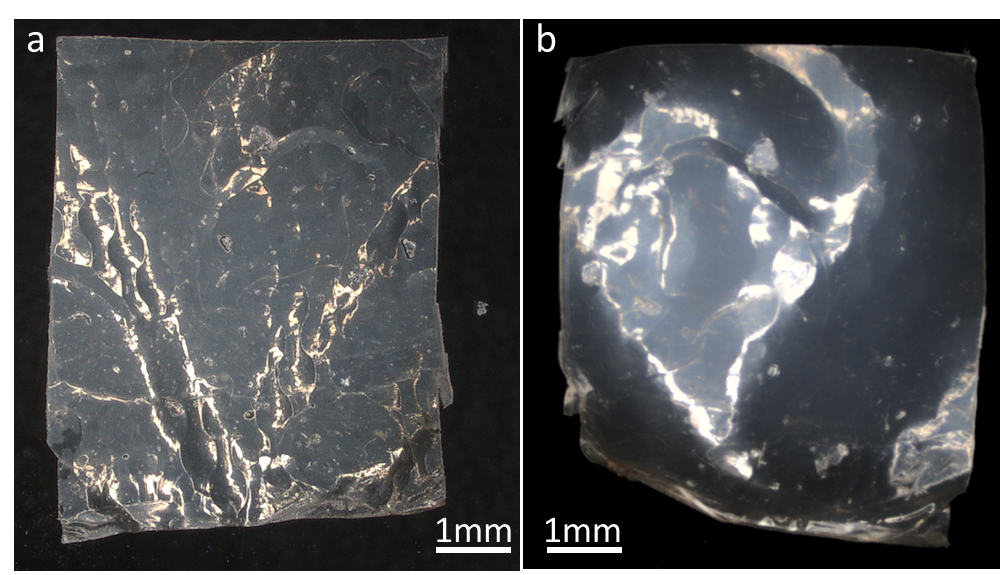

Supplement: Supplementary file 1 — Supplementary Figures. [file 41598_2022_12931_MOESM1_ESM.docx]
